# Risk factors for prolonged virus shedding of respiratory tract and fecal in adults with severe acute respiratory syndrome coronavirus‐2 infection

**DOI:** 10.1002/jcla.23923

**Published:** 2021-08-13

**Authors:** Shun Zhang, Hui Zhu, Honghua Ye, Yaoren Hu, Nanhong Zheng, Zuoan Huang, Zi Xiong, Liyun Fu, Ting Cai

**Affiliations:** ^1^ Department of Experimental Medical Science HwaMei Hospital University of Chinese Academy of Sciences Ningbo China; ^2^ Key Laboratory of Diagnosis and Treatment of Digestive System Tumors of Zhejiang Province Ningbo China; ^3^ Department of Cardiology HwaMei Hospital University of Chinese Academy of Sciences Ningbo China; ^4^ Department of Infection and Hepatology HwaMei Hospital University of Chinese Academy of Sciences Ningbo China

**Keywords:** COVID‐19, longitudinal study, prolonged viral shedding, risk factor, SARS‐CoV‐2

## Abstract

**Background:**

The dynamic alteration and comparative study of severe acute respiratory syndrome coronavirus 2 (SARS‐CoV‐2) RNA shedding pattern during treatment are limited. This study explores the potential risk factors influencing prolonged viral shedding in COVID‐19.

**Methods:**

A total of 126 COVID‐19 patients were enrolled in this retrospective longitudinal study. A multivariate logistic regression analysis was carried out to estimate the potential risk factors.

**Results:**

38.1% (48/126) cases presented prolonged respiratory tract viral shedding, and 30 (23.8%) cases presented prolonged rectal swab viral shedding. Obesity (*OR*, 3.31; 95% CI, 1.08–10.09), positive rectal swab (*OR*, 3.43; 95% CI, 1.53–7.7), treatment by lopinavir/ritonavir with chloroquine phosphate (*OR*, 2.5; 95% CI, 1.04–6.03), the interval from onset to antiviral treatment more than 7 days (*OR*, 2.26; 95% CI, 1.04–4.93), lower CD4+ T cell (*OR*, 0.92; 95% CI, 0.86–0.99) and higher NK cells (*OR*, 1.11; 95% CI, 1.02–1.20) were significantly associated with prolonged respiratory tract viral shedding. CD3−CD56+ NK cells (*OR*, 0.87; 95% CI, 0.76–0.99) were related with prolonged fecal shedding.

**Conclusions:**

Obesity, delayed antiviral treatment, and positive SARS‐CoV‐2 for stool were independent risk factors for prolonged SARS‐CoV‐2 RNA shedding of the respiratory tract. A combination of LPV/r and abidol as the initial antiviral regimen was effective in shortening the duration of viral shedding compared with LPV/r combined with chloroquine phosphate. CD4+ T cell and NK cells were significantly associated with prolonged viral shedding, and further studies are to be warranted to determine the mechanism of immunomodulatory response in virus clearance.

## Graphical abstract

The multivariable logistic regression analysis suggested that BMI (*OR*, 3.32; 95% CI, 1.08–10.0), positive rectal swab (*OR*, 3.43; 95% CI, 1.53–7.7), treatment by lopinavir/ritonavir with chloroquine phosphate (*OR*, 2.5; 95% CI, 1.04–6.03), interval from onset to antiviral treatment more than 7 days (*OR*, 2.26; 95% CI, 1.04–4.93), lower CD4+ T cell (*OR*, 0.92; 95% CI, 0.86–0.99) and higher NK cells (*OR*, 1.11; 95% CI, 1.02–1.20) were significantly associated with prolonged respiratory tract viral shedding even after adjustment for age and gender. Additionally, CD3−CD56+ NK cells (*OR*, 0.87; 95% CI: 0.76–0.99) on admission were linked to prolonged fecal shedding.

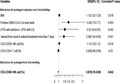

## 1 INTRODUCTION

Severe acute respiratory syndrome coronavirus 2 (SARS‐CoV‐2) results in the coronavirus disease 2019 (COVID‐19) pandemic.^1^ The clinical and epidemiological characteristics of COVID‐19 and the structure of SARS‐CoV‐2 have been elucidated gradually.^2^, ^3^, ^4^, ^5^ Although people are generally susceptible to SARS‐CoV‐2, approximately 80% of COVID‐19 are mild.^6^ However, many cases were reported persistent positive for respiratory SARS‐CoV‐2 nucleic acid, even after acute exudative lesions in lungs were almost absorbed, and symptoms were completely relieved.^7^ Some studies showed that only a small proportion of patients have gastrointestinal (GI) manifestation, not all the fecal positive patients presented GI manifestation, and SARS‐CoV‐2 infection and replication persisted in the GI tract even after viral RNA undetectable in the respiratory sample.^8^, ^9^ Some studies found that older age, obesity, and the lack of lopinavir/ritonavir (LPV/r) treatment were independent risk factors for prolonged respiratory viral shedding.^10^, ^11^ The relations among viral shedding duration in respiratory and fecal simultaneously, clinical parameters, and treatment efficacy of COVID‐19 are still elusive.

Thus, we performed a longitudinal retrospective study of 126 hospitalized laboratory‐confirmed COVID‐19 patients to evaluate the risk factors associated with prolonged viral shedding, to optimize the antiviral treatment options, and to explore the relationship between the respiratory tract and fecal viral shedding duration.

## 2 MATERIALS AND METHODS

### 2.1 Study design and participants

This longitudinal retrospective study enrolled 126 patients who were older than 18 years old and had available SARS‐CoV‐2 RNA detection data in HwaMei Hospital, University of Chinese Academy of Sciences, Zhejiang, China between January 23, 2020 and April 7, 2020. Patients were diagnosed based on the diagnosis and treatment protocols from the National Health Commission of the People's Republic of China.^12^ The final follow‐up was up to June 15, 2020.

This study was approved by the Ethics Committee of HwaMei Hospital, University of Chinese Academy of Sciences (NO. PJ‐NBEY‐KY‐2020–008–01). Oral informed consents were obtained from all subjects. The procedures were following the ethical standards of the Helsinki Declaration.

The privacy rights of human subjects are always observed.

### 2.2 Data collection

Demographic and clinical data were obtained from electronic medical records. The discharge criteria and the duration of temperature have been delineated.^7^

Cases were confirmed by quantitative real‐time reverse transcription‐polymerase chain reaction (qRT‐PCR) assay for nasopharyngeal/throat swabs or sputum, and the quantification cycle (*C*q) results including two targeting genes: open reading frame (ORF) 1a/1b and nuclear (N), less than 40 was defined as a positive result.^2^ Respiratory tract specimens were collected and detected every other day during hospitalization, and repeat QRT‐PCR testing on 3rd, 5th, 7th, 14th, 21th, and 28th days after discharge were conducted.

All confirmed cases were offered treatment, and the antiviral drugs mainly included LPV/r (400 mg/100 mg, orally, bid), arbidol (200 mg, orally, TID), chloroquine phosphate (CQ, 500 mg, orally, bid), and alpha‐interferon (5 million U, inhalation, QD). All data were double‐checked by two physicians.

### 2.3 Cutoff of respiratory and fecal viral shedding

The duration of viral shedding was measured as the time from symptom onset till the time when nuclei acid tested negative twice consecutively (sample collection interval of at least 1 day), without turning positive thereafter. A report by time‐dependent respiratory tract viral testing among 802 initial‐testing‐positive patients revealed that 50% of patients turned negative in 28 days.^13^ 60 patients with both positive viral RNA test for respiratory tract and rectal swab specimens simultaneously showed that the duration of viral shedding was 28.5 days (IQR, 21.25–38) for respiratory tract and 27.5 days (IQR, 18.3–39) for rectal swabs. Thus, the cutoff of the prolonged respiratory tract or fecal viral shedding was determined as the duration of SARS‐CoV‐2 RNA positive for more than 28 days in this study.

### 2.4 Statistical analysis

Continuous variables were presented median and inter‐quartile range (IQR), and the differences between groups were evaluated with the Mann‐Whitney *U* test. Categorical variables were presented as frequency and percentages, and proportions for categorical variables were compared using chi‐square tests or Fisher's exact test. Univariate and adjusted multivariate logistic regression analyses were carried out to estimate the potential risk factors. Spearman's correlation analysis was performed to identify the relationship between the duration of the respiratory tract and fecal viral shedding. All statistical analyses above were performed by IBM SPSS statistics version 24.0. (IBM, Armonk, New York, USA). The forest plot diagram was visualized using the ggplot2 and forest plot packages for R software (version 4.0.0). Two‐sided *p* < 0.05 was defined as statistical significance.

## 3 RESULTS

### 3.1 Demographic and clinical manifestations

A total of 126 hospitalized patients with COVID‐19 were included, and 38.1% (48/126) of patients presented the duration of respiratory tract viral shedding >28 days (Table 1). Of these patients, 82 (65.1%) were women and the median age was 50 (IQR, 35.8–58.3), and 29 (23.0%) had one or more comorbidities. The median body mass index (BMI) was 23.5 kg/m^2^ (IQR, 20.9–26.0), and cases with a duration of respiratory tract viral shedding >28 days showed a significantly higher percentage of obesity (BMI >28 kg/m^2^) than the <28 days cases (20.8% vs 9%).

**TABLE 1:** Characteristics of 126 Hospitalized Patients with SARS‐CoV‐2 infection

Characteristics	Group	All patients, 126 (%)	Duration of viral shedding of the respiratory tract		*p*
			<28 days, 78(%)	≥28 days, 48(%)	
Age (years)	Median, IQR	50 (35.8–58.3)	50 (32–58.3)	49.5 (38–58.8)	0.575
	<50	61 (48.4)	37 (47.4)	24 (50.0)	0.856
	≥50	64 (50.8)	40 (51.3)	24 (50.0)	
Gender (%)	Male	44 (34.9)	24 (30.8)	20 (41.7)	0.250
	Female	82 (65.1)	54 (69.2)	28 (58.3)	
BMI (kg/m^2^)	Median, IQR	23.5 (20.9–26)	23.4 (20.4–25.3)	24.5 (21.6–27.5)	0.030
	18.5–24	66 (52.4)	46 (59.0)	20 (41.7)	0.061
	<18.5	6 (4.8)	5 (6.4)	1 (2.1)	
	24–28	36 (28.6)	19 (24.4)	17 (35.4)	
	>28	17 (13.5)	7 (9.0)	10 (20.8)	
Epidemiologic history (%)	No	25 (19.8)	14 (17.9)	11 (22.9)	0.646
	Yes	101(80.2)	64 (82.1)	37 (77.1)	
Fever (%)	No	42 (33.3)	28 (35.9)	14 (29.2)	0.560
	Yes	84 (66.7)	50 (64.1)	34 (70.8)	
Nasal congestion (%)	No	116 (92.1)	71 (91.0)	45 (93.8)	0.741
	Yes	10 (7.9)	7 (9.0)	3 (6.3)	
Cough (%)	No	64 (50.8)	40 (51.3)	24 (50.0)	>0.99
	Yes	62 (49.2)	38 (48.7)	24 (50.0)	
Chest pain and stuffiness (%)	No	118 (93.7)	74 (94.9)	44 (91.7)	0.709
	Yes	8 (6.3)	4 (5.1)	4 (8.3)	
Fatigue (%)	No	101 (80.2)	63 (80.8)	38 (79.2)	>0.99
	Yes	25 (19.8)	15 (19.2)	10 (20.8)	
Diarrhea (%)	No	115 (91.3)	72 (92.3)	43 (89.6)	0.747
	Yes	11 (8.7)	6 (7.7)	5 (10.4)	
Chronic hepatitis B (%)	No	120 (95.2)	75 (96.2)	45 (93.8)	0.673
	Yes	6 (4.8)	3 (3.8)	3 (6.3)	
Coronary heart disease (%)	No	124 (98.4)	77 (98.7)	47 (97.9)	>0.99
	Yes	2 (1.6)	1 (1.3)	1 (2.1)	
Hypertension (%)	No	105 (83.3)	62 (79.5)	43 (89.6)	0.218
	Yes	21 (16.7)	16 (20.5)	5 (10.4)	
Diabetes (%)	No	118 (93.7)	74 (94.9)	44 (91.7)	0.709
	Yes	8 (6.3)	4 (5.1)	4 (8.3)	
Smoking (%)	No	116 (92.1)	72 (92.3)	44 (91.7)	>0.99
	Yes	10 (7.9)	6 (7.7)	4 (8.3)	
Positive SARS‐CoV‐2 for stool (%)	Negative	66 (52.4)	49 (62.8)	17 (35.4)	0.008
	<28 days	30 (23.8)	16 (53.3)	14 (46.7)	
	>28 days	30 (23.8)	13 (43.3)	17 (56.7)	
Clinical classification (%)	Mild	15 (11.9%)	8 (53.3)	7 (46.7)	0.479
	Moderate	106 (84.1%)	68 (64.1)	38 (35.9)	
	Severe	5 (4.0%)	2 (40.0)	3 (60.0)	
Chest CT scan (%)	Unilateral pneumonia	59 (46.8)	34 (43.6)	25 (52.1)	0.365
	Bilateral pneumonia	67 (53.2)	44 (56.4)	23 (47.9)	

Note

Data are presented as the median and inter‐quartile range (IQR) and *n* (%).

*p* values comparing the duration of viral shedding of <28 days and >28 days are from Mann‐Whitney *U* test, chi‐square test, and Fisher's exact test.

Abbreviations: BMI, body mass index; CT, computed tomography.

Epidemiological data showed that there were 101(80.2%) patients who had closely contacted with confirmed COVID‐19 patients, and most cases were temple gathering and familial cluster.^14^ The most common symptoms at the onset of illness were fever (84 [66.7%]), cough (62 [42.9%]), and fatigue (25 [19.8%]). 15 (11.9%), 106 (84.1%), and 5 (4.0%) patients were categorized into the group of mild, moderate, and severe types, respectively. Additionally, 60 (47.6%) cases were presented with positive viral RNA test for rectal swab, and 30 (23.8%) cases presented the duration of fecal samples viral shedding for more than 28 days (Table 1). For chest CT scans, more than half of the patients (67, 53.2%) showed bilateral pneumonia on admission.

### 3.2 Laboratory findings and immunological indicators

Baseline laboratory parameters were showed in Table S1, and the T lymphocyte subsets showed that the level of NK cells in the respiratory prolonged group (>28 days) was significantly higher than those in the non‐prolonged group (<28 days) on admission (*p* = 0.022). However, no significant difference was discovered in other laboratory findings between the two groups. The median *C*q values on admission were 32.3 (IQR 28.3–36.3) for ORF1ab and 31.7 (IQR 28.1–35.4) for N‐gene, and there was no significant difference in baseline *C*q values between the respiratory shedding prolonged and non‐prolonged groups (*p* ˃ 0.05) (Table S1).

### 3.3 Treatment and outcome

All patients received antiviral therapy, and treatment was initiated at a median of 3 days (IQR, 1–7.3) from symptom onset (Table 2). Just 15.1% (19/126) of patients received systemic corticosteroid treatment. Based on the therapeutic regimen, 37(29.4%) patients were treated by LPV/r with arbidol and 72 (57.1%) by LPV/r with CQ. The median duration of respiratory tract SARS‐CoV‐2 shedding and the hospital stay in the non‐prolonged group (<28 days) were 19 days (IQR, 14.5–22.5) and 14 days (IQR, 12–18), respectively, which were significantly shorter than those in the prolonged group (>28 days) of 37.5 days (IQR, 32–46.8) and 21 days (IQR, 16.5–26.8) (*p* < 0.05) (Table 2).

**TABLE 2:** Treatment and outcomes of COVID‐19 patients

Variables	Group	All patients, 126 (%)	Duration of viral shedding		*p*
			<28 days, 78 (%)	≥28 days, 48 (%)	
**Treatment**					
The interval from the onset of symptoms to antiviral therapy (days)		3 (1–7.3)	3 (1–6.3)	4 (1–9)	0.034
Antiviral regimen (%)	LPV/r with arbidol	37 (29.4)	27 (34.6)	10 (20.8)	0.040
	LPV/r with CQ	72 (57.1)	37 (47.4)	35 (72.9)	
Systemic corticosteroid treatment (%)	No	107 (84.9)	67 (85.9)	40 (83.3)	0.799
	Yes	19 (15.1)	11 (14.1)	8 (16.7)	
**Outcomes**					
Duration of viral shedding on the anal swab species (days)		27.5 (18.3–39)	24 (15.5–32.5)	30 (20–40)	0.214
Duration of hospital length of stay (days)		16 (13–21)	14 (12–18)	21 (16.5–26.8)	<0.001
Duration of viral shedding on respiratory tract samples (days)		24 (17.5–34)	19 (14.5–22.5)	37.5 (32–46.8)	<0.001

Note

Data are presented as the median and inter‐quartile range (IQR) and *n* (%).

*p* values comparing the duration of viral shedding of <28 days and >28 days are from the Mann‐Whitney *U* test and chi‐square test.

Abbreviations: CQ, chloroquine phosphate; LPV/r, lopinavir/ritonavir.

**FIGURE 1:**
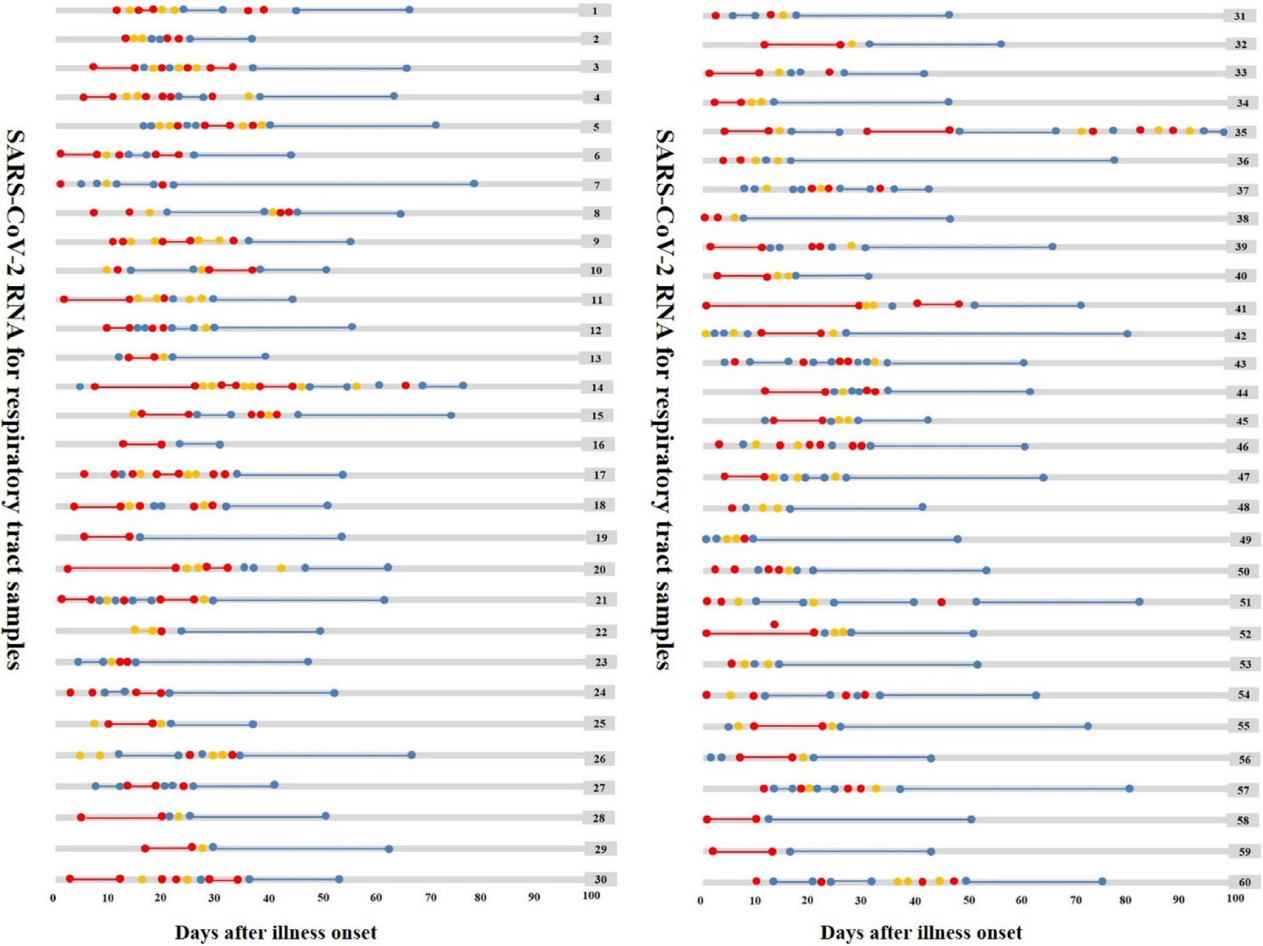
Dynamic changes in SARS‐CoV‐2 RNA for respiratory tract samples since illness onset. SARS‐CoV‐2 negative results are represented by blue dots, invalid results by yellow dots, and positive results are in red dots, plotted on a time scale from the date of the first test since illness onset

**FIGURE 2:**
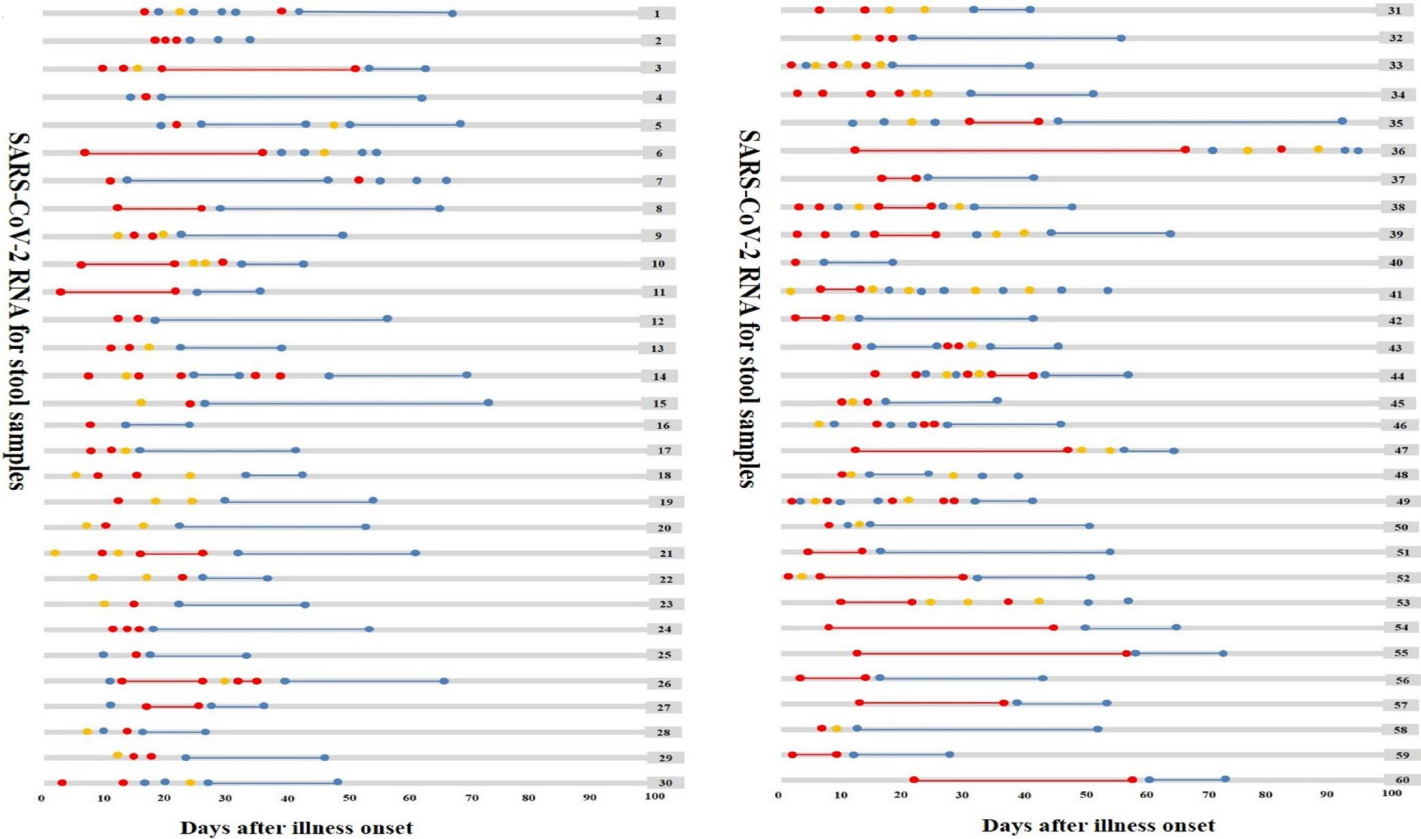
Dynamic changes in SARS‐CoV‐2 RNA for stool samples since illness onset. SARS‐CoV‐2 negative results are represented by blue dots, invalid results by yellow dots, and positive results are in red dots, plotted on a time scale from the date of the first test since illness onset

We identified 60 patients with both positive viral RNA tests for respiratory tract and rectal swab specimens simultaneously (Figures 1 and 2). It was worth mentioning that two patients, patient 35 and patient 36, had the duration of viral shedding of SARS‐CoV‐2 more than 90 days. Patient 35 was initially positive of respiratory virus and converted to negative at day 14 after onset, but later, reverted to positive from day 32 to day 48 and positive at day 75, day 84, and day 90 (Figure 1). The detection for rectal swab samples showed a conversion from an initial SARS‐CoV‐2 negative result to positive and back to negative in 12 days (Figure 2). Patient 36 repeatedly tested positive via rectal swab specimens for 68 days since onset, then test negative at day 72, suspected positive at day 78, positive again at day 84, suspected positive at day 90, and negative at day 96and 98 (Figure 2). However, the respiratory viral shedding time in patient 36 was just 12 days (Figure 1).

### 3.4 Risk factors with prolonged respiratory tract viral shedding

The univariable analysis and multivariable logistic regression analysis were presented in Table 3 and Table S2. The multivariable logistic regression analysis suggested that obesity (BMI >28) (*OR*, 3.31; 95% CI, 1.08–10.09), positive detection of rectal swab species for SARS‐CoV‐2 RNA (*OR*, 3.43; 95% CI, 1.53–7.7), treatment by LPV/r with CQ (*OR*, 2.5; 95% CI, 1.04–6.03), the prolonged interval time of more than 7 days from illness onset to antiviral treatment (*OR*, 2.26; 95% CI, 1.04–4.93), CD4+ T cell (*OR*, 0.92; 95% CI, 0.86–0.99), and NK cells (*OR*, 1.11; 95% CI, 1.02–1.20) were significantly associated with prolonged viral shedding of respiratory tract even after the adjustment for age and gender.

**TABLE 3:** Multivariable logistic regression analysis of factors associated with prolonged shedding of SARS‐CoV‐2 RNA among patients with COVID‐19

Variables	Crude *OR* (95% CI)	*p*	Adjusted *OR* (95% CI)	*p*
**Risk factors for prolonged respiratory tract viral shedding**				
BMI	1.13 (1.02–1.25)	0.015	1.13 (1.02–1.25)	0.016
18.5–24	Reference	Reference	Reference	Reference
<18.5	0.46 (0.05–4.19)	0.491	0.41 (0.04–3.87)	0.438
24–28	2.06 (0.89–4.76)	0.092	1.98 (0.85–4.63)	0.113
>28	3.29 (1.09–9.86)	0.034	3.31 (1.08–10.09)	0.036
Positive SARS‐CoV‐2 on anal swab	3.08 (1.46–6.52)	0.003	3.43 (1.53–7.7)	0.003
LPV/r with arbidol vs. LPV/r with chloroquine phosphate	2.55 (1.08–6.04)	0.033	2.50 (1.04–6.03)	0.042
Interval from onset to antiviral treatment >7 days	2.22 (1.03–4.80)	0.043	2.26 (1.04–4.93)	0.041
CD3−CD56+ NK cell (%)	1.1 (1.02–1.18)	0.016	1.11 (1.02–1.2)	0.016
CD3+CD4+ T cell (%)	0.93 (0.88–0.99)	0.043	0.92 (0.86–0.99)	0.030
**Risk factor for prolonged fecal viral shedding**				
CD3−CD56+ NK cell (%)	0.93 (0.85–1.03)	0.161	0.87 (0.76–0.99)	0.042

Note

Univariate and adjusted multivariate logistic regression analysis were carried out to estimate the potential risk factors associated with prolonged duration of SARS‐CoV‐2 RNA shedding, and the age and sex were adjusted as covariates in adjusted model.

Abbreviations: BMI, body mass index; LPV/r, lopinavir/ritonavir.

### 3.5 Risk factors with prolonged fecal viral shedding

Patients with positive detection of rectal swab species for SARS‐CoV‐2 accounted for almost 47.6% (60/126), and the white blood cell count (*OR*, 1.281; 95% CI, 1.030–1.593), hs‐CRP (*OR*, 0.975; 95% CI, 0.952–0.999), CD3+ CD8+ T cells (*OR*, 0.9; 95% CI, 0.812–0.997;), and CD4/CD8 ratio (*OR*, 2.016; 95% CI, 1.037–3.920) on admission were significantly associated with positive viral shedding in rectal swab species after adjustment for age and gender (Figure S1 and Table S3). Additionally, CD3−CD56+ NK cells (*OR*, 0.87; 95% CI: 0.76–0.99) on admission were linked to prolonged fecal shedding (Table 3 and Table S4). However, Spearman's correlation analysis showed no significant correlation between the duration of the respiratory tract and fecal viral shedding among 60 patients with both positive SARS‐CoV‐2 RNA in the respiratory tract and fecal specimens simultaneously (*r* = 0.218, *p* = 0.095).

## 4 DISCUSSION

The viral shedding window of COVID‐19 remains largely uncharacterized, which poses challenges to reappraising discontinuation of quarantine. In our study, the dynamic detection of SARS‐CoV‐2 RNA on the respiratory tract and rectal swabs among 126 infected cases was conducted. We found that 38.1% (48/126) of confirmed cases presented prolonged respiratory tract viral shedding, and 23.8% (30/126) of cases presented prolonged fecal viral shedding. Obesity, positive detection of rectal swab species for viral RNA, delayed antiviral treatment, elevated NK, and decreased CD4+ T cell cells were significantly related to prolonged respiratory viral shedding, and patients treated by LPV/r combined with arbidol had remarkably shortened duration of respiratory viral shedding compared with those treated by LPV/r combined with CQ. We also identified that decreased CD3−CD56+ NK cells on admission were related to shorten fecal shedding.

In this study, the median duration from onset of symptoms to antiviral therapy was 3 days (IQR, 1–7.3 days), and the interval from onset to antiviral treatment more than 7 days was an independent risk factor for prolonged viral shedding. Our results were in line with the current report that timely detection and supportive care for symptomatic patients with COVID‐19 contributed to the clearance of viral RNA.^15^ Previous data indicated the complex interaction between obesity and increased pneumonia risk, and the underlying mechanism might involve immune system dysregulation.^16^, ^17^ A retrospective cohort study in French reported that the proportion of COVID‐19 patients who required invasive mechanical ventilation increased with BMI.^18^ Additionally, a previous study on influenza A H1N1 found obesity was associated with prolonged hospitalization and the need for IMV.^19^ In our cohort, the obesity rate was 13.5%, and obesity was an independent predisposition factor for prolonged respiratory tract viral RNA shedding after adjustment for age and gender. Obesity‐related low‐grade inflammation appeared to be associated with the prognosis of virus infection, and further study is to be warranted to elucidate etiological mechanisms.

No specific antiviral drugs have been approved to be safe and effective for the treatment of COVID‐19 so far. Some drugs with antiviral properties and immunomodulatory effects were officially recommended for COVID‐19, including LPV/r, arbidol, CQ, etc.^12^, ^20^ LPV/r and arbidol were previously recommended for patients with SARS and MERS‐CoV.^21^, ^22^ Some studies implied that the use of LPV/r and arbidol was associated with an apparent favorable clinical response to COVID‐19,^23^, ^24^ but controversy existed in different studies. For example, Cheng and his/her colleagues reported LPV/r had no advantage in shortening the duration of SARS‐CoV‐2 shedding in five mild patients in Taiwan.^25^ The US Center for Disease Control clarified that CQ inhibited viral replication by interfering with SARS‐CoV‐2 binding to the ACE2 receptor.^26^ CQ has been used to treat COVID‐19 with no benefits having been observed in a multinational registry analysis.^27^ Our findings demonstrated that the use of LPV/r in combination with arbidol was linked with a shorter hospital stay and duration of viral shedding compared with the use of LPV/r in combination with CQ.

T lymphocyte plays a vital role in SARS‐CoV‐2 elimination by stimulating antigen‐(Ag) specific T lymphocytes.^28^ Emerging data indicated that lymphocytopenia was one of the important predictor factors influencing exacerbation and mortality of patients with COVID‐19.^29^, ^30^, ^31^ Likewise, our data identified that decreased CD4+ T cell was a significant risk factor for prolonged viral shedding of the respiratory tract. NK cells were one of the predominant lymphocyte subsets and accounted for the third of intrahepatic lymphocytes.^32^ Viral infection‐induced NK cells present activated and mature phenotypes with stronger cytotoxic capabilities in a murine model.^33^ However, our study showed the increased NK cell was a risk factor for prolonged viral shedding of the respiratory tract but a protective factor for fecal viral shedding. Our study also showed that 60 (47.6%) of 126 COVID‐19 patients detected positive fecal viral results, and 30 (23.8%) presented prolonged fecal viral shedding, and the positive SARS‐CoV‐2 for stool was related to prolonged duration of respiratory viral shedding. However, GI symptoms just occurred in 11(8.7%) of patients in our study. Likewise, a meta‐analysis reported that approximately 7.4% of patients with COVID‐19 experienced diarrhea, and about 30% to 50% of patients were detected for positive SARS‐CoV‐2 RNA.^34^ Chen et al.^15^ found diarrhea was independently associated with prolonged SARS‐CoV‐2 RNA shedding in the respiratory tract among 267 confirmed cases. It has been proven that ACE2 is abundantly expressed in the GI tract.^35^, ^36^ Positive detection of SARS‐CoV‐2 in digestive tract specimens suggested that pathogenicity of SARS‐CoV‐2 might be transmitted by the fecal‐oral route, not limited by droplet transmission.^37^ In our study, the viral shedding patterns of the respiratory and digestive tract were not consistent. Similarly, Wang et al.^38^ reported that the median duration of positive SARS‐CoV‐2 in fecal samples was 25 days, significantly longer than that in respiratory tract samples by approximately 9 days and that patients with SARS‐CoV‐2 RNA in fecal presented higher proportions of complete absorption and no lesion progression of chest CT results.

There were some limitations in this study. First, most patients enrolled in this study had mild clinical symptoms, so our findings may not apply to severe and critical patients. Second, immune indicators including T lymphocyte subsets and cytokines were not conducted in all patients, so the role of immunomodulatory effects in eliminating SARS‐CoV‐2 might be underestimated. Third, our study was a single‐center study and limited by a relatively small sample size. Fourth, the underlying reason for the unparallel detection results between the respiratory tract and fecal samples needs to be clarified in the future.

In conclusion, obesity, delayed antiviral treatment, and positive SARS‐CoV‐2 for stool were independent risk factors for prolonged SARS‐CoV‐2 RNA shedding of the respiratory tract. A combination of LPV/r and abidol as the initial antiviral regimen was effective in shortening the duration of viral shedding compared with LPV/r combined with CQ. Decreased CD4+ T cell and elevated CD3−CD56+ NK cells were associated with prolonged respiratory tract RNA shedding, while decreased CD3−CD56+ NK cells might be linked with shortened fecal RNA shedding, and further studies are to be warranted to determine the mechanism of immunomodulatory response in virus clearance.

## CONFLICT OF INTEREST

None.

## AUTHOR CONTRIBUTIONS

Ting Cai and Liyun Fu designed the study. Shun Zhang and Hui Zhu analyzed the data and drafted the article. Honghua Ye, Yaoren Hu, Nanhong Zheng, Zuoan Huang, and Zi Xiong contributed to the acquisition of subjects and data. Hui Zhu and Liyun Fu contributed to the analysis and interpretation of data. Ting Cai has primary responsibility for the final content.

## Supporting information

Fig S1

Tab S1

Tab S2

Tab S3

Tab S4

## Data Availability

The original anonymized data are available on request from the corresponding author.
