# Supplementary figures and images for: Risk factors for prolonged virus shedding of respiratory tract and fecal in adults with severe acute respiratory syndrome coronavirus‐2 infection
Source: J Clin Lab Anal. 2021 Aug 13;35(9):e23923. doi: 10.1002/jcla.23923 (PMC8418473; doi:10.1002/jcla.23923)

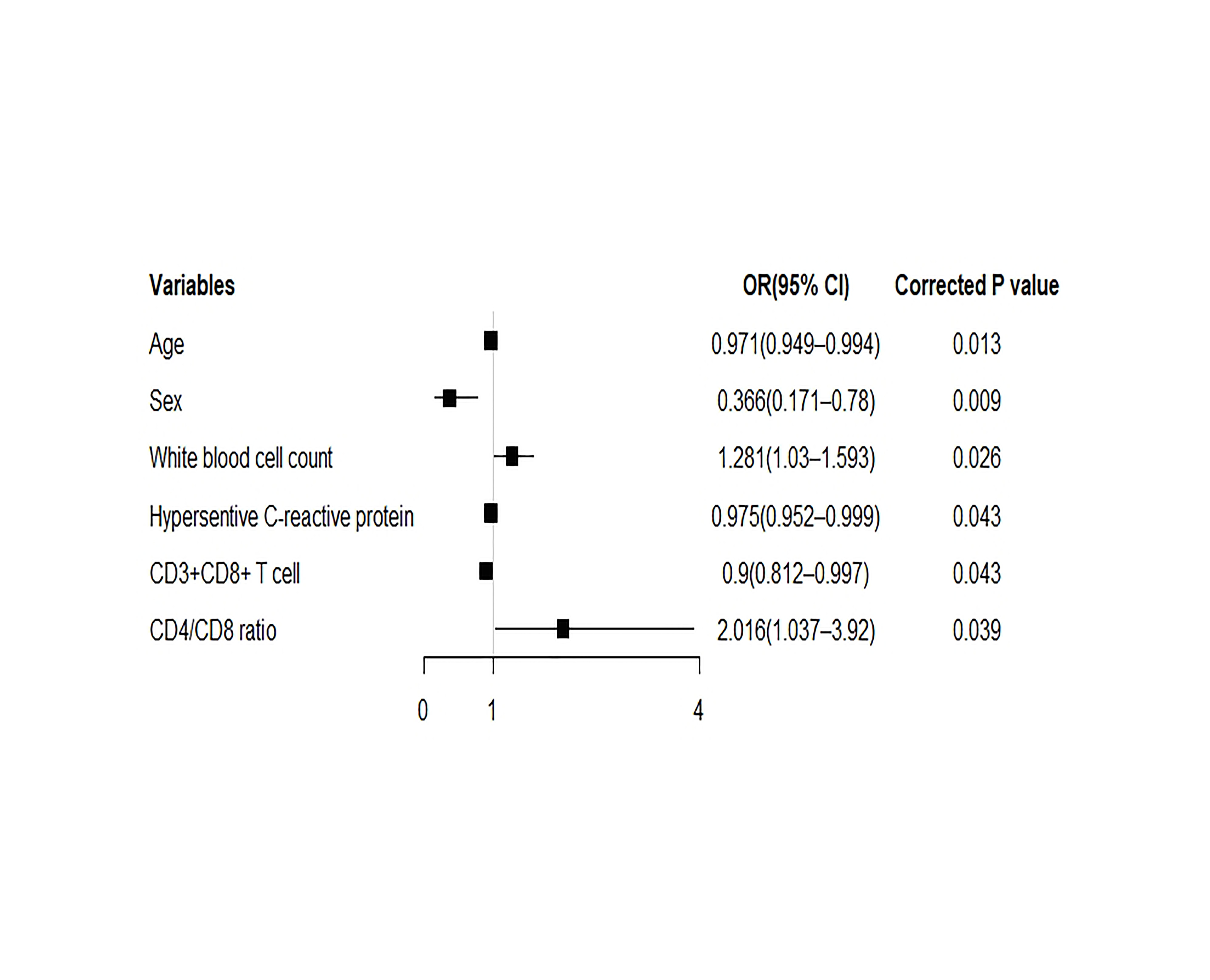

Supplement: Supplementary file 1 — Fig S1 [file JCLA-35-e23923-s003.tif]
