# Supplementary material for: Risk factors for prolonged virus shedding of respiratory tract and fecal in adults with severe acute respiratory syndrome coronavirus‐2 infection
Source: J Clin Lab Anal. 2021 Aug 13;35(9):e23923. doi: 10.1002/jcla.23923 (PMC8418473; doi:10.1002/jcla.23923)
Supplement: Supplementary file 2 — Tab S1 [file JCLA-35-e23923-s005.docx]

**Supplementary Table1 Laboratory findings of 126 Hospitalized Patients with SARS-CoV-2 infection on admission**

| Laboratory findings | Group | All patients, | Duration of viral shedding | | *P* |
| --- | --- | --- | --- | --- | --- |
|  |  | 126 (%) | <28 days, 78(%) | ≥28 days, 48(%) |  |
| White blood cell count (10^9^/L) |  | 5.0 (4-6.4) | 4.8 (3.8-6.2) | 5.3 (4.2-7) | 0.085 |
|  | <4 | 30 (23.8) | 22 (28.2) | 8 (16.7) | 0.112 |
|  | 4-10 | 92 (73) | 55 (70.5) | 37 (77.1) |  |
|  | >10 | 4 (3.2) | 1 (1.3) | 3 (6.3) |  |
| Platelet count (10^9^/L) |  | 204 (155.8-255.5) | 202 (154.8-246.8) | 206.5 (160.3-273) | 0.444 |
|  | <150 | 25 (19.8) | 17 (21.8) | 8 (16.7) | 0.504 |
|  | >150 | 101 (80.2) | 61 (78.2) | 40 (83.3) |  |
| Hemoglobin (g/L) |  | 133 (123-141.3) | 131 (121-140.3) | 136 (126.5-145.5) | 0.105 |
| Lymphocyte count (10^9^/L) |  | 1.2 (0.9-1.6) | 1.2 (0.9-1.7) | 1.2 (0.9-1.6) | 0.982 |
|  | <1 | 41 (32.5) | 27 (34.6) | 14 (29.2) | 0.562 |
|  | >1 | 85 (67.5) | 51 (65.4) | 34 (70.8) |  |
| hs-CRP (mg/L) |  | 7.7 (1.9-18.6) | 8.1 (2-15.6) | 6.9 (1.5-26.2) | 0.741 |
|  | <6 | 54 (42.9) | 33 (42.3) | 21 (43.8) | >0.99 |
|  | >6 | 67 (53.2) | 42 (53.8) | 25 (52.1) |  |
| Procalcitonin (ng/mL) | <0.5 | 110 (87.3) | 67 (85.9) | 43 (89.6) | 0.523 |
|  | >0.5 | 2 (1.6) | 2 (2.6) | 0 (0) |  |
| Lactose dehydrogenase (U/L) |  | 208.5 (173.8-241.8) | 208.5 (173.5-247) | 208 (173.3-237.5) | 0.874 |
|  | <250 | 97 (77) | 60 (76.9) | 37 (77.1) | >0.99 |
|  | >250 | 29 (23) | 18 (23.1) | 11 (22.9) |  |
| Aspartate aminotransferase (U/L) |  | 23 (17-28) | 22 (17-28.3) | 24 (16-28) | 0.676 |
|  | <40 | 114 (90.5) | 71 (91) | 43 (89.6) | >0.99 |
|  | >40 | 12 (9.5) | 7 (9) | 5 (10.4) |  |
| Alanine aminotransferase (U/L) |  | 21 (14-31) | 20 (13-28.8) | 22 (14.8-34) | 0.218 |
|  | <40 | 105 (83.3) | 66 (84.6) | 39 (81.3) | 0.806 |
|  | >40 | 21 (16.7) | 12 (15.4) | 9 (18.8) |  |
| Total bilirubin (mmol/L) |  | 8.9 (6.7-13.8) | 8.5 (6.6-14) | 9.8 (6.7-13.6) | 0.736 |
|  | <17.1 | 107 (84.9) | 66 (84.6) | 41 (85.4) | >0.99 |
|  | >17.1 | 19 (15.1) | 12 (15.4) | 7 (14.6) |  |
| Creatine kinase (U/L) |  | 76 (53.5-95.3) | 79 (54-104.3) | 73 (48-90) | 0.494 |
|  | <200 | 122 (96.8) | 74 (94.9) | 48 (100) | 0.162 |
|  | >200 | 4 (3.2) | 4 (5.1) | 0 (0) |  |
| Creatinine (μmol/L) |  | 56.8 (48.3-68.6) | 56.4 (48.1-67.4) | 61.1 (48.6-69.7) | 0.324 |
|  | <80 | 115 (91.3) | 73 (93.6) | 42 (87.5) | 0.331 |
|  | >80 | 11 (8.7) | 5 (6.4) | 6 (12.5) |  |
| D-dimer (mg/L) |  | 100 (82-159.5) | 100 (73.5-130.5) | 104.5 (94.8-177.3) | 0.202 |
|  | <500 | 103 (81.7) | 65 (83.3) | 38 (79.2) | >0.99 |
|  | >500 | 5 (4) | 3 (3.8) | 2 (4.2) |  |
| Urea (mmol/L) |  | 4.2 (3.3-5) | 3.9 (3.1-4.7) | 4.4 (3.8-5.1) | 0.013 |
| Sodium (mmol/L) |  | 138.4 (137-140.3) | 138.3 (137-140.4) | 138.6 (136.8-139.9) | 0.862 |
| Potassium (mmol/L) |  | 3.9 (3.6-4.2) | 3.9 (3.5-4.2) | 4 (3.7-4.4) | 0.045 |
| Chloride (mmol/L) |  | 101.5 (99.4-103.6) | 101.6 (99.7-103.8) | 101.2 (98.6-103.6) | 0.39 |
| IL-2 (pg/ml) |  | 0.9 (0.5-1.9) | 0.7 (0.5-1.5) | 1 (0.6-2.1) | 0.062 |
| IL-4 (pg/ml) |  | 1.9 (1.2-2.6) | 2 (1.5-2.6) | 1.8 (1-2.4) | 0.194 |
| IL-6 (pg/ml) |  | 4.1 (1.9-11.8) | 3.8 (1.8-9.1) | 4.8 (2.2-13.8) | 0.170 |
| IL-10 (pg/ml) |  | 3.1 (2.2-4.6) | 2.9 (2-4.6) | 3.3 (2.2-4.7) | 0.433 |
| TNF-α (pg/ml) |  | 1.4 (1-1.7) | 1.4 (1.1-1.7) | 1.5 (0.9-1.8) | 0.672 |
| TFN-γ (pg/ml) |  | 1.3 (0.9-1.7) | 1.2 (0.9-1.6) | 1.3 (0.9-1.9) | 0.266 |
| CD3+ T cell (%) |  | 69.3 (64.7-75.5) | 71.3 (66.6-76.7) | 66.4 (62.7-72.3) | 0.055 |
| CD45RA+CD45RO+ T cell (%) |  | 1.3 (0.8-1.8) | 1.28 (0.8-1.8) | 1.3 (0.8-1.9) | 0.714 |
| CD3-CD56+ NK cell (%) |  | 13.8 (9.2-19.6) | 12.1 (8.5-16.6) | 17.9 (9.6-25.2) | 0.022 |
| CD19+ B-cells (%) |  | 11.5 (8-15.5) | 12.08 (7.79-17.26) | 11.47 (8.1-14.7) | 0.891 |
| CD3+CD4+ T cell (%) |  | 40.3 (36-46.5) | 42.77 (37.6-50.5) | 39.7 (32.7-43.9) | 0.069 |
| CD4/CD8 T-cell ratio (%) |  | 2 (1.5-3.0) | 2.1 (1.6-3.39) | 1.9 (1.4-2.8) | 0.268 |
| CD3+CD25+ T cell (%) |  | 7.2 (5.8-8.8) | 6.61(5.03-8.57) | 7.3 (6.1-9.3) | 0.348 |
| CD3+DR+ T cell (%) |  | 12.3 (9.5-17.5) | 14.51(10.42-17.69) | 11.4 (8.5-17.2) | 0.255 |
| CD8+DR+ T cell (%) |  | 5.7 (4.1-8.2) | 5.74 (4.16-8.01) | 5.6 (3.7-9.0) | 0.912 |
| CD4+CD25+ T cell (%) |  | 5.4 (4.2-7.5) | 5.2 (3.9-7.2) | 6.64 (4.9-7.7) | 0.111 |
| CD3+CD8+ T cell (%) |  | 20.5 (14.7-24.8) | 19.8 (14.5-25.0) | 21.1 (14.8-24.3) | 0.835 |
| CD8+CD38+ T cell (%) |  | 2.7 (1.9-3.6) | 2.6 (1.55-3.7) | 2.9 (2.1-3.6) | 0.425 |
| CD4+CD45RA+/  CD4+CD45RA+62L+ T cell (%) | | 25.4(20.8-31.2) | 25.9 (22.1-31.3) | 25.3 (18.1-29.0) | 0.364 |
| CD4+CD45RA-/  CD4+CD45RO+ T cell (%) | | 34.7(26.9-39.9) | 34.4 (24.3-41.2) | 34.9 (29.0-39.5) | 0.729 |
| ORF1ab (*C*q value) |  | 32.3 (28.3-36.3) | 32.1 (28.4-36.3) | 33.1 (27.2-36.3) | 0.799 |
| N-gene (*C*q value) |  | 31.7 (28.1-35.4) | 31.4 (27.6-35.3) | 31.7 (28.8-36.1) | 0.613 |

hs-CRP, hypersensitive C-reactive protein; ORF, open reading frame; N gene, Nuclear gene; *C*q, quantification cycle.

Data are presented as median and inter-quartile range (IQR) and n (%).

*P* values comparing duration of viral shedding of <28 days and >28 days are from Mann-Whitney *U* test, *χ*² test and Fisher’s exact test.
