# Supplementary material for: Risk factors for prolonged virus shedding of respiratory tract and fecal in adults with severe acute respiratory syndrome coronavirus‐2 infection
Source: J Clin Lab Anal. 2021 Aug 13;35(9):e23923. doi: 10.1002/jcla.23923 (PMC8418473; doi:10.1002/jcla.23923)
Supplement: Supplementary file 3 — Tab S2 [file JCLA-35-e23923-s002.docx]

**Supplementary Table 2 Multivariable logistic regression analyses of factors associated with** **prolonged respiratory tract viral shedding in 126 hospitalized patients**

| Variables | Crude *OR* (95%*CI*) | *P* | Adjusted *OR* (95%*CI*) | *P* |
| --- | --- | --- | --- | --- |
| Gender | 0.622(0.29-1.32) | 0.214 |  |  |
| Age | 1.008(0.985-1.031) | 0.49 |  |  |
| BMI | 1.13(1.02-1.25) | 0.015 | 1.13(1.02-1.25) | 0.016 |
| 18.5-24 | Reference | 0.076 | Reference | 0.079 |
| <18.5 | 0.46(0.05-4.19) | 0.491 | 0.41(0.04-3.87) | 0.438 |
| 24-28 | 2.06(0.89-4.76) | 0.092 | 1.98(0.85-4.63) | 0.113 |
| >28 | 3.29(1.09-9.86) | 0.034 | 3.31(1.08-10.09) | 0.036 |
| Bilateral pneumonia | 0.71(0.35-1.46) | 0.354 | 0.68(0.32-1.44) | 0.309 |
| Fever | 1.36(0.63-2.95) | 0.437 | 1.56(0.7-3.49) | 0.280 |
| Nasal congestion | 0.68(0.17-2.75) | 0.585 | 0.78(0.19-3.27) | 0.735 |
| Cough | 1.05(0.51-2.16) | 0.889 | 1.03(0.5-2.12) | 0.945 |
| Chest pain and stuffiness | 1.68(0.4-7.06) | 0.478 | 1.89(0.44-8.13) | 0.394 |
| Fatigue | 1.11(0.45-2.71) | 0.827 | 0.95(0.37-2.42) | 0.911 |
| Diarrhea | 1.4(0.4-4.85) | 0.600 | 1.21(0.34-4.33) | 0.769 |
| Chronic hepatitis B | 1.67(0.32-8.61) | 0.542 | 1.41(0.27-7.52) | 0.687 |
| Coronary heart disease | 1.64(0.1-26.82) | 0.729 | 1.25(0.07-22.63) | 0.882 |
| Hypertension | 0.45(0.15-1.32) | 0.147 | 0.36(0.11-1.15) | 0.085 |
| Diabetes | 1.68(0.4-7.06) | 0.478 | 1.57(0.36-6.78) | 0.547 |
| Smoking | 1.09(0.29-4.08) | 0.897 | 0.68(0.16-2.93) | 0.607 |
| Systemic corticosteroid treatment | 1.22(0.45-3.28) | 0.696 | 1.09(0.39-3.05) | 0.870 |
| Positive SARS-CoV-2 on rectal swab | 3.08(1.46-6.52) | 0.003 | 3.43(1.53-7.7) | 0.003 |
| Clinical classification | 0.96(0.38-2.41) | 0.929 | 0.90(0.35-2.30) | 0.826 |
| LPV/r with CQ vs. LPV/r with arbidol | 2.55(1.08-6.04) | 0.033 | 2.5(1.04-6.03) | 0.042 |
| Epidemiologic exposure | 0.736(0.303-1.787) | 0.50 | 0.72(0.29-1.78) | 0.477 |
| Interval time from onset to antiviral treatment more than 7 days | 2.22(1.03-4.80) | 0.043 | 2.26(1.04-4.93) | 0.041 |
| CD3+ T cell (%) | 0.95(0.9–1.01) | 0.093 | 0.95(0.9–1.01) | 0.093 |
| CD45RA+CD45RO+ T cell (%) | 1.13(0.62–2.04) | 0.697 | 1.14(0.63–2.09) | 0.662 |
| CD3-CD56+ NK cell (%) | 1.1(1.02–1.18) | 0.016 | 1.11(1.02–1.2) | 0.016 |
| CD19+ B-cells (%) | 1(0.92–1.09) | 0.977 | 1.01(0.92–1.09) | 0.901 |
| CD3+CD4+ T cell (%) | 0.93(0.88–0.99) | 0.043 | 0.92(0.86–0.99) | 0.030 |
| CD4/CD8 T-cell ratio (%) | 0.81(0.51–1.29) | 0.377 | 0.71(0.39–1.28) | 0.255 |
| CD3+CD25+ T cell (%) | 1.08(0.88–1.32) | 0.475 | 1.07(0.87–1.32) | 0.503 |
| CD3+DR+ T cell (%) | 0.98(0.92–1.06) | 0.674 | 0.98(0.91–1.06) | 0.640 |
| CD8+DR+ T cell (%) | 1(0.86–1.17) | 0.957 | 1.01(0.86–1.17) | 0.945 |
| CD4+CD25+ T cell (%) | 1.23(0.96–1.57) | 0.108 | 1.23(0.95–1.58) | 0.111 |
| CD3+CD8+ T cell (%) | 1(0.93–1.08) | 0.936 | 1.01(0.92–1.1) | 0.868 |
| CD8+CD38+ T cell (%) | 1.04(0.83–1.29) | 0.741 | 1.03(0.83–1.29) | 0.787 |
| CD4+CD45RA+/ CD4+CD45RA+62L+ T cell (%) | 0.97(0.92–1.03) | 0.335 | 0.97(0.91–1.03) | 0.347 |
| CD4+CD45RA-/ CD4+CD45RO+ T cell (%) | 1.01(0.96–1.06) | 0.780 | 1.01(0.96–1.06) | 0.795 |
| IL-2 | 1.43(0.9–2.26) | 0.132 | 1.47(0.92–2.34) | 0.110 |
| IL-4 | 0.74(0.48–1.15) | 0.183 | 0.75(0.48–1.18) | 0.214 |
| IL-6 | 1.01(0.99–1.04) | 0.401 | 1.01(0.99–1.04) | 0.385 |
| IL-10 | 1.01(0.94–1.09) | 0.808 | 1.01(0.94–1.1) | 0.748 |
| TNF-α | 1.14(0.64–2.03) | 0.655 | 1.15(0.64–2.09) | 0.635 |
| TFN-γ | 1.34(0.87–2.07) | 0.186 | 1.33(0.85–2.07) | 0.207 |

BMI, body mass index; LPV/r, Lopinavir/ritonavir; CQ, chloroquine phosphate.

Univariate and adjusted multivariate logistic regression analyses were carried out to estimate the potential risk factors associated with prolonged duration of SARS-CoV-2 RNA shedding, and the age and sex were adjusted as covariates in the adjusted model.
