# Supplementary material for: Risk factors for prolonged virus shedding of respiratory tract and fecal in adults with severe acute respiratory syndrome coronavirus‐2 infection
Source: J Clin Lab Anal. 2021 Aug 13;35(9):e23923. doi: 10.1002/jcla.23923 (PMC8418473; doi:10.1002/jcla.23923)
Supplement: Supplementary file 4 — Tab S3 [file JCLA-35-e23923-s004.docx]

**Supplementary Table 3 Multivariable logistic regression analyses of factors associated with positive rectal swab samples for SARS-CoV-2 RNA**

| Variables | Crude *OR* (95%CI) | *P* | Adjusted *OR* (95%*CI*) | *P* |
| --- | --- | --- | --- | --- |
| Age | 0.971(0.949–0.994) | 0.013 |  |  |
| Gender | 0.366(0.171–0.78) | 0.009 |  |  |
| BMI | 1.056(0.964–1.157) | 0.239 | 1.055(0.957–1.163) | 0.280 |
| 18.5-24 | Reference | 0.524 | Reference | 0.563 |
| <18.5 | 0.929(0.145–5.929) | 0.938 | 0.798(0.107–5.965) | 0.826 |
| 24-28 | 1.557(0.689–3.516) | 0.287 | 1.698(0.713–4.045) | 0.232 |
| >28 | 1.99(0.675–5.865) | 0.212 | 1.722(0.546–5.434) | 0.354 |
| Bilateral pneumonia | 0.784(0.389–1.581) | 0.496 | 1.051(0.491–2.25) | 0.897 |
| Epidemiologic exposure | 0.981(0.408–2.357) | 0.966 | 1.278(0.499–3.273) | 0.610 |
| Fever | 0.563(0.266–1.189) | 0.132 | 0.594(0.265–1.33) | 0.205 |
| Nasal congestion | 1.722(0.462–6.426) | 0.418 | 1.616(0.402–6.496) | 0.499 |
| Cough | 0.725(0.359–1.462) | 0.368 | 0.678(0.321–1.431) | 0.308 |
| Pain and stuffiness | 0.642(0.147–2.81) | 0.556 | 0.571(0.124–2.624) | 0.471 |
| Fatigue | 0.834(0.346–2.012) | 0.686 | 0.549(0.206–1.464) | 0.231 |
| Diarrhea | 0.602(0.167–2.169) | 0.438 | 0.552(0.144–2.12) | 0.387 |
| Coronary heart disease | / | 0.999 | / | 0.999 |
| Chronic hepatitis B | 1.105(0.214–5.697) | 0.905 | 0.85(0.151–4.795) | 0.854 |
| Hypertension | 0.627(0.24–1.638) | 0.341 | 1.23(0.417–3.626) | 0.707 |
| Diabetes | 1.909(0.436–8.356) | 0.391 | 2.797(0.586–13.34) | 0.197 |
| Smoking | 0.714(0.191–2.665) | 0.616 | 0.36(0.081–1.613) | 0.182 |
| Systemic corticosteroid treatment | 0.594(0.217–1.626) | 0.311 | 0.7(0.233–2.102) | 0.525 |
| Duration of temperature recovery (days) | 0.983(0.939–1.029) | 0.466 | 0.989(0.943–1.038) | 0.665 |
| LPV/r with arbidol vs. LPV/r with chloroquine phosphate | 2.328(1.016–5.336) | 0.046 | 1.873(0.781–4.492) | 0.160 |
| White blood cell count | 1.347(1.092–1.663) | 0.005 | 1.281(1.03–1.593) | 0.026 |
| Platelet count | 1.001(0.996–1.006) | 0.777 | 1(0.995–1.005) | 0.976 |
| Hemoglobin | 1.038(1.011–1.066) | 0.005 | 1.023(0.992–1.054) | 0.143 |
| Lymphocyte count | 1.515(0.823–2.79) | 0.182 | 1.274(0.824–1.97) | 0.276 |
| Hs-CRP | 0.972(0.951–0.993) | 0.009 | 0.975(0.952–0.999) | 0.043 |
| Procalcitonin, ng/mL | 1.2(0.073–19.676) | 0.898 | 1.015(0.059–17.344) | 0.992 |
| Lactose dehydrogenase | 0.992(0.986–0.998) | 0.012 | 0.995(0.988–1.002) | 0.144 |
| Aspartate aminotransferase | 0.997(0.976–1.018) | 0.759 | 0.997(0.977–1.018) | 0.798 |
| Alanine aminotransferase | 1.009(0.991–1.027) | 0.344 | 1.002(0.982–1.023) | 0.815 |
| Total bilirubin | 1.02(0.967–1.077) | 0.462 | 1.001(0.942–1.063) | 0.985 |
| Creatine kinase | 1.002(0.995–1.009) | 0.604 | 1.002(0.995–1.01) | 0.488 |
| Creatinine | 1.019(0.995–1.045) | 0.125 | 1.003(0.973–1.034) | 0.856 |
| D–dimer | 1(0.999–1.002) | 0.741 | 1(0.999–1.002) | 0.726 |
| IL-2 | 1.192(0.786–1.809) | 0.408 | 1.299(0.804–2.098) | 0.285 |
| IL-4 | 0.724(0.471–1.111) | 0.139 | 0.805(0.514–1.261) | 0.343 |
| IL-6 | 0.995(0.972–1.019) | 0.700 | 1.005(0.98–1.03) | 0.723 |
| IL-10 | 0.983(0.909–1.064) | 0.675 | 1.023(0.942–1.109) | 0.593 |
| TNF-α | 0.912(0.515–1.615) | 0.753 | 1.108(0.605–2.028) | 0.740 |
| TFN-γ | 1.07(0.815–1.406) | 0.626 | 1.037(0.824–1.306) | 0.757 |
| CD3+ T cell | 1.024(0.972–1.079) | 0.375 | 1.021(0.964–1.081) | 0.471 |
| CD45RA+CD45RO+ T cell | 0.869(0.478–1.578) | 0.644 | 0.895(0.482–1.662) | 0.725 |
| CD3-CD56+ NK cell | 1.008(0.946–1.075) | 0.801 | 1.004(0.933–1.08) | 0.920 |
| CD19+ B-cell | 0.973(0.896–1.055) | 0.505 | 0.992(0.909–1.083) | 0.854 |
| CD3+CD4+ T cell | 1.016(0.957–1.078) | 0.610 | 1.059(0.984–1.139) | 0.126 |
| CD4/CD8 T-cell ratio | 1.102(0.704–1.724) | 0.672 | 2.016(1.037–3.92) | 0.039 |
| CD3+CD25+ T cell | 0.898(0.729–1.106) | 0.312 | 0.885(0.711–1.1) | 0.271 |
| CD3+DR+ T cell | 0.949(0.882–1.022) | 0.167 | 0.954(0.884–1.031) | 0.236 |
| CD8+DR+ T cell | 0.936(0.805–1.089) | 0.394 | 0.953(0.815–1.115) | 0.549 |
| CD4+CD25+ T cell | 0.885(0.696–1.125) | 0.319 | 0.843(0.653–1.089) | 0.191 |
| CD3+CD8+ T cell | 0.985(0.918–1.058) | 0.684 | 0.9(0.812–0.997) | 0.043 |
| CD8+CD38+ T cell | 0.933(0.75–1.162) | 0.537 | 0.914(0.727–1.149) | 0.439 |
| CD4+CD45RA+/ CD4+CD45RA+62L+ T cell | 0.98(0.925–1.038) | 0.488 | 0.958(0.896–1.025) | 0.215 |
| CD4+CD45RA-/ CD4+CD45RO+ T cell | 1.03(0.977–1.087) | 0.273 | 1.037(0.981–1.096) | 0.204 |

BMI, body mass index; LPV/r, Lopinavir/ritonavir.

Univariate and adjusted multivariate logistic regression analyses were carried out to estimate the potential risk factors associated with prolonged duration of SARS-CoV-2 RNA shedding, and the age and sex were adjusted as covariates in the adjusted model.
