# Supplementary material for: Risk factors for prolonged virus shedding of respiratory tract and fecal in adults with severe acute respiratory syndrome coronavirus‐2 infection
Source: J Clin Lab Anal. 2021 Aug 13;35(9):e23923. doi: 10.1002/jcla.23923 (PMC8418473; doi:10.1002/jcla.23923)
Supplement: Supplementary file 5 — Tab S4 [file JCLA-35-e23923-s001.docx]

**Supplementary Table 4 Multivariable logistic regression analyses of factors associated with prolonged viral shedding of SARS-CoV-2 RNA in fecal samples**

| Variables | Crude *OR* (95%*CI*) | P | Adjusted *OR* (95%*CI*) | P |
| --- | --- | --- | --- | --- |
| Age | 1.01(0.98–1.04) | 0.665 |  |  |
| Gender | 0.76(0.28–2.11) | 0.605 |  |  |
| BMI | 0.96(0.85–1.08) | 0.534 | 0.97(0.86–1.09) | 0.572 |
| 18.5-24 | Reference |  | Reference |  |
| <18.5 | / | / | / | / |
| 24-28 | 0.51(0.15–1.66) | 0.262 | 0.49(0.15–1.63) | 0.245 |
| >28 | 1.3(0.3–5.64) | 0.726 | 1.4(0.31–6.23) | 0.660 |
| Fever | 0.57(0.2–1.62) | 0.294 | 0.6(0.2–1.77) | 0.356 |
| Nasal congestion | 0.17(0.02–1.58) | 0.119 | 0.18(0.02–1.73) | 0.138 |
| Cough | 1.97(0.7–5.54) | 0.197 | 2.05(0.72–5.84) | 0.177 |
| Chest pain and stuffiness | 2.07(0.18–24.15) | 0.561 | 2.65(0.21–33.15) | 0.451 |
| Fatigue | 0.8(0.22–2.97) | 0.739 | 0.71(0.18–2.79) | 0.629 |
| Diarrhea | 3.22(0.32–32.89) | 0.324 | 3.06(0.29–32.6) | 0.354 |
| Hypertension | 0.56(0.12–2.57) | 0.452 | 0.49(0.1–2.53) | 0.396 |
| Diabetes | 0.64(0.1–4.15) | 0.643 | 0.61(0.09–4.06) | 0.612 |
| Smoking | 3.22(0.32–32.89) | 0.324 | 2.84(0.24–33.67) | 0.408 |
| Clinical classification | 0.64(0.17–2.42) | 0.506 | 0.64(0.17–2.48) | 0.519 |
| Bilateral pneumonia | 0.44(0.16–1.25) | 0.124 | 0.42(0.14–1.23) | 0.113 |
| LPV/r with chloroquine phosphate vs. LPV/r with arbidol | 2(0.51–7.78) | 0.317 | 2.04(0.52–8.03) | 0.309 |
| Epidemiologic exposure | 1.52(0.42–5.47) | 0.520 | 1.49(0.4–5.5) | 0.553 |
| Interval time from onset to antiviral treatment more than 7 days | 1.06(0.92–1.22) | 0.411 | 1.08(0.93–1.24) | 0.326 |
| White blood cell count | 1.1(0.93–1.3) | 0.256 | 1.11(0.92–1.33) | 0.270 |
| Platelet count | 1(0.99–1.01) | 0.206 | 1.01(1–1.01) | 0.133 |
| Hemoglobin | 1.01(0.98–1.04) | 0.614 | 1.01(0.97–1.05) | 0.624 |
| Lymphocyte count | 1.2(0.77–1.86) | 0.412 | 1.25(0.72–2.17) | 0.428 |
| hs-CRP | 0.99(0.96–1.03) | 0.685 | 0.99(0.96–1.02) | 0.617 |
| Lactose dehydrogenase | 1(0.99–1.02) | 0.336 | 1.01(1–1.02) | 0.315 |
| Aspartate aminotransferase | 1.01(0.98–1.04) | 0.502 | 1.01(0.98–1.04) | 0.450 |
| Alanine aminotransferase | 0.66(0.18–2.36) | 0.520 | 0.65(0.17–2.4) | 0.514 |
| Total bilirubin | 1.03(0.95–1.11) | 0.473 | 1.03(0.94–1.12) | 0.525 |
| Creatine kinase | 1(0.99–1.01) | 0.606 | 1(0.99–1.01) | 0.612 |
| creatinine | 0.99(0.96–1.02) | 0.480 | 0.97(0.92–1.01) | 0.160 |
| IL-2 | 1.07(0.89–1.29) | 0.471 | 1.07(0.88–1.3) | 0.480 |
| IL-4 | 1.68(0.83–3.39) | 0.147 | 1.72(0.84–3.52) | 0.141 |
| IL-6 | 1.04(0.98–1.1) | 0.174 | 1.04(0.98–1.09) | 0.187 |
| IL-10 | 0.97(0.83–1.13) | 0.656 | 0.96(0.81–1.13) | 0.594 |
| TNF-α | 2.18(0.79–6.02) | 0.130 | 2.2(0.79–6.14) | 0.132 |
| TFN-γ | 1.11(0.81–1.53) | 0.527 | 1.12(0.81–1.56) | 0.480 |
| CD3+ T cell (%) | 1.07(0.98–1.18) | 0.132 | 1.13(1–1.28) | 0.054 |
| CD45RA+CD45RO+ T cell (%) | 1.26(0.52–3.01) | 0.610 | 1.26(0.52–3.08) | 0.610 |
| CD3-CD56+ NK cell (%) | 0.93(0.85–1.03) | 0.161 | 0.87(0.76–0.99) | 0.042 |
| CD19+ B-cells (%) | 0.99(0.89–1.11) | 0.912 | 1.02(0.9–1.15) | 0.777 |
| CD3+CD4+ T cell (%) | 1.03(0.94–1.13) | 0.517 | 1.03(0.93–1.13) | 0.591 |
| CD4/CD8 T-cell ratio (%) | 0.9(0.5–1.61) | 0.724 | 0.63(0.29–1.39) | 0.253 |
| CD3+CD25+ T cell (%) | 0.92(0.69–1.22) | 0.573 | 0.94(0.7–1.24) | 0.646 |
| CD3+DR+ T cell (%) | 1.03(0.93–1.14) | 0.592 | 1.01(0.91–1.12) | 0.825 |
| CD8+DR+ T cell (%) | 1.03(0.84–1.26) | 0.813 | 0.98(0.79–1.22) | 0.871 |
| CD4+CD25+ T cell (%) | 0.9(0.66–1.22) | 0.503 | 0.95(0.69–1.3) | 0.734 |
| CD3+CD8+ T cell (%) | 1.05(0.95–1.17) | 0.324 | 1.18(1–1.41) | 0.053 |
| CD8+CD38+ T cell (%) | 0.96(0.67–1.4) | 0.846 | 0.91(0.62–1.34) | 0.641 |
| CD4+CD45RA+/ CD4+CD45RA+62L+ T cell (%) | 0.99(0.92–1.06) | 0.732 | 1.01(0.93–1.09) | 0.879 |
| CD4+CD45RA-/ CD4+CD45RO+ T cell (%) | 1.06(0.97–1.15) | 0.183 | 1.04(0.96–1.14) | 0.320 |

BMI, body mass index; LPV/r, Lopinavir/ritonavir.

Univariate and adjusted multivariate logistic regression analyses were carried out to estimate the potential risk factors associated with prolonged duration of SARS-CoV-2 RNA shedding, and the age and sex were adjusted as covariates in the adjusted model.
